# Congenital Uterine Anomalies and Pregnancy Outcomes: A Population-Based Retrospective Cohort Study

**DOI:** 10.3390/jcm15176788

**Published:** 2026-09-01

**Authors:** Ahyoung Choi, Min-A Kim, Geum Joon Cho, Han Sung Hwang

**Affiliations:** 1 Department of Obstetrics and Gynecology, Gangnam Severance Hospital, Yonsei University College of Medicine, Seoul 06273, Republic of Korea; cay423@yuhs.ac (A.C.); makim302@yuhs.ac (M.-A.K.); 2 Department of Obstetrics and Gynecology, Korea University Guro Hospital, Korea University College of Medicine, Seoul 08308, Republic of Korea; 3 Department of Obstetrics and Gynecology, Konkuk University School of Medicine, Seoul 05030, Republic of Korea

**Keywords:** congenital uterine anomaly, Müllerian anomaly, pregnancy outcome, preterm birth, Cesarean delivery, small for gestational age

## Abstract

**Background/Objectives**: Congenital uterine anomalies (CUAs) are associated with adverse pregnancy outcomes, but large-scale data, particularly in Asian populations, remain limited. We evaluated obstetric and neonatal outcomes in women with CUAs using a large national Korean cohort. **Methods**: This population-based retrospective cohort study used the Korean National Health Insurance Service database and included 878,000 singleton deliveries between 2018 and 2023. CUAs were identified using International Classification of Disease, 10th Revision (ICD-10) codes and categorized as double, septate, bicornuate, or unicornuate uterus. Outcomes were compared with women without CUA using multivariable logistic regression adjusted for maternal age, parity, chronic hypertension, and pregestational diabetes mellitus. **Results**: CUAs were identified in 6038 women (0.68%), most commonly double uterus. Risks were higher for Cesarean delivery (adjusted odds ratio [aOR] 2.57, 95% confident interval [CI] 2.38–2.77), breech presentation (6.42, 5.91–6.99), placenta previa (1.79, 1.45–2.22), and placental abruption (3.01, 2.26–4.01). Neonates were more likely to be preterm (2.43, 2.10–2.80), small for gestational age (1.90, 1.70–2.10), and to experience respiratory distress syndrome (2.04, 1.63–2.55) and intraventricular hemorrhage (3.04, 2.00–4.62). **Conclusions**: CUAs are associated with increased risks of adverse obstetric and neonatal outcomes, with subtype-specific variations, warranting tailored prenatal counseling and management.

## 1. Introduction

Congenital uterine anomaly (CUA), also known as Müllerian anomaly, refers to a structural abnormality of the uterus. The female reproductive system develops from the Müllerian ducts (paramesonephric ducts) during early fetal development [1]. Aberrations in this developmental sequence can lead to CUA. The human uterus develops through a tightly coordinated sequence of Mullerian duct elongation, midline fusion of the paired ducts, and subsequent resorption of the midline septum. Disruption at any of these stages gives rise to the distinct categories of uterine anomaly [1,2]. There are several types of CUAs. The most widely accepted classification of CUAs is based on the American Society for Reproductive Medicine (ASRM) classification system [2]. The ASRM 2021 classification categorizes Müllerian anomalies into nine groups: Müllerian agenesis, cervical agenesis, unicornuate uterus, uterus didelphys, bicornuate uterus, septate uterus, longitudinal vaginal septum, transverse vaginal septum, and complex anomalies.

Several classification systems have been proposed over time, including the earlier American Fertility Society system and European Society of Human Reproduction and Embryology/European Society for Gynecological Endoscopy classification, in addition to the ASRM 2021 system [2]. The coexistence of multiple classification systems and diagnostic modalities used has contributed to substantial heterogenicity in the reported prevalence and distribution of anomaly subtypes across studies.

CUAs occur due to genetic factors or environmental effects. Some cases may be associated with other congenital anomalies, including renal anomalies [3]. The exact prevalence of CUA in the general population is unknown; however, some researchers have reported that about 5.5% of an unselected population have CUA [4]. The prevalence of CUA in Korea is not precisely known due to a lack of data, but a previous study reported a prevalence of 0.075% [5]. The most common type of uterine anomaly was uterine didelphys (30.6%), followed by bicornuate uterus (19.4%). More than 40% of patients with CUA were asymptomatic and diagnosed incidentally, so over 80% of cases were found in adulthood.

Reported prevalence estimates vary widely, ranging from approximately 0.06% to as high as 38% [4,6,7,8,9,10,11]. The variation reflects differences in the populations studied, such as unselected women compared with those who have infertility or recurrent pregnancy loss, as well as differences in the diagnostic modality applied. In unselected populations the pooled prevalence has been estimated at around 5.5%, whereas it rises substantially among women with recurrent miscarriage [4]. This wide variation underscores the value of large, unselected, population-based data for estimating the true burden of disease.

The prevalence of CUA is higher in women with infertility or a history of miscarriage or preterm delivery [12]. While many women with CUA can conceive and deliver a full-term baby, CUA is associated with menstrual symptoms, infertility, and recurrent pregnancy loss or preterm delivery [13,14]. CUA may increase the risk of complications such as fetal growth restriction, malpresentation, and obstructed labor [15].

Mechanistically, the adverse reproductive and obstetric consequences of uterine anomalies are thought to arise from a reduced and often distorted uterine cavity, diminished myometrial mass, and impaired uterine vascular supply, which together may compromise implantation, placentation, and the capacity or the uterus to accommodate the growing fetus [13,16,17]. These structural constraints provide a plausible biological basis for the associations between CUA and preterm birth, malpresentation, abnormal placentation, and impaired fetal growth.

Increased evaluation and examination lead to many women with infertility being diagnosed with CUA. The incidence rate of CUA in women with infertility is higher than in the unselected population. The number of women with CUA undergoing assisted reproductive technology (ART) and the conception rate among women with CUA are increasing [18]. Previous studies have reported that pregnancies in women with CUA may have increased rates of preterm labor, preterm premature rupture of membranes, fetal malpresentation, fetal growth restriction, and Cesarean delivery [19,20]. However, those studies were limited by modest sample sizes due to the rarity of uterine anomalies.

Despite this growing clinical relevance, the existing literature is dominated by relatively small, single-centered studies with heterogeneous populations and classification systems, and large, population-based data from Asian cohorts remain scarce [13,19]. Therefore, there is a need for a study with a large sample size, particularly in a Korean population. The objective of this study was to use a large national database to comprehensively characterized obstetric and neonatal outcomes in women diagnosed with CUA in Korea, overall and by anatomical subtype.

## 2. Materials and Methods

### 2.1. Study Design and Data Source

This population-based retrospective cohort study was conducted using data from the Korean National Health Insurance Service (NHIS), a mandatory, single-payer system that provides comprehensive health coverage for approximately 97% of the Korean population. The NHIS database contains longitudinal information on demographic characteristics, medical diagnoses, procedures, and prescriptions, all coded according to the International Classification of Diseases, 10th Revision (ICD-10). The database has been widely used and validated in epidemiologic studies of pregnancy outcomes. The NHIS database has been well characterized in previous data resource profiles and has been widely used and validated in epidemiologic research, including studies of perinatal and pregnancy outcomes [21,22,23]. Nevertheless, to our knowledge the accuracy of the ICD-10 codes used to identify CUA specifically has not been formally validated within this database, and the diagnoses were not confirmed by imaging or surgery in the present study. We therefore relied on clinically recorded diagnostic codes, and the resulting potential for exposure misclassification is addressed in the Discussion Section.

### 2.2. Study Population

We identified all women who delivered between 1 January 2018 and 31 December 2023. Among 1,293,752 deliveries during the study period, we excluded multiple gestations to eliminate the confounding effects of plurality on pregnancy outcomes. We further excluded cases with missing information on key variables, including maternal age, birth weight, and gestational age, as well as cases with implausible birth weight values. After applying these criteria, a total of 878,000 singleton pregnancies were included in the final analysis. All data were de-identified prior to analysis.

Specifically, we sequentially excluded 57,338 multiple gestations, 354,638 pregnancies with missing maternal age, birth weight, or gestational age, 2158 with an implausible birth weight, and 1618 with other uterine anomalies (Q51.0 and Q51.5–Q51.9), leaving 878,000 singleton pregnancies. Implausible birth weight was defined as a birth weight of 0 kg or less, or 8 kg or more. The stepwise selection of the study population, including the number of records excluded at each step, is summarized in the study flow diagram (Figure S1). Among the 878,000 singleton deliveries, 486,911 were to nulliparous women, and 91,406 women delivered two or more times during the study period. To account for this non-independence, a subgroup analysis restricted to nulliparous women, in which each woman contributed a single pregnancy, was also performed (Table S2).

### 2.3. Exposure: Congenital Uterine Anomalies

CUAs were identified based on ICD-10 diagnostic codes recorded before or during pregnancy. To reflect clinically meaningful anatomical subtypes, uterine anomalies were categorized into four mutually exclusive groups: double uterus (Q51.1 or O34.0), unicornuate uterus (Q51.2), bicornuate uterus (Q51.3), and septate uterus (Q51.4). All remaining uterine anomalies within the Q50–Q52 code range, excluding the above subtypes, were grouped as “other uterine anomalies.” Women without any recorded diagnosis of uterine anomalies served as the reference group.

A diagnostic code recorded at any time before or during the index pregnancy was considered sufficient for classification, in order to capture anomalies incidentally outside of pregnancy as well as those identified during antenatal care. Because the claims database dose not record the diagnostic modality (e.g., three-dimensional ultrasonography, magnetic resonance imaging, hysteroscopy, or laparoscopy) used to establish each diagnosis, these codes reflect clinically recorded diagnoses rather than imaging.

### 2.4. Outcome Measures

The primary maternal outcomes included pregnancy-induced hypertension (PIH), gestational diabetes mellitus (GDM), placenta previa, placental abruption, postpartum hemorrhage (PPH), and Cesarean delivery. These outcomes were identified using ICD-10 codes recorded during the delivery hospitalization. Neonatal outcomes included preterm birth (<37 weeks of gestation), birth weight, small for gestational age (SGA; birth weight below the 10th percentile for gestational age), and large for gestational age (LGA; above the 90th percentile). We further evaluated neonatal morbidities, including transient tachypnea of the newborn (TTN), respiratory distress syndrome (RDS), necrotizing enterocolitis (NEC), intraventricular hemorrhage (IVH), and bronchopulmonary dysplasia (BPD).

Maternal outcomes were confirmed from ICD-10 codes recorded during the delivery admission, including PIH (O13–O16), GDM (O24), placenta previa (O44), placental abruption (O45), PPH (O72), Cesarean delivery (O82) and breech presentation (O32). Neonatal morbidities were defined using ICD-10 codes for TTN (P22.1), RDS (P22.0), NEC (P77), IVH (P52), and BPD (P27.1). SGA were defined relative to the Korean sex and gestational age-specific birth weight reference [24]. There are no established core outcome sets specifically for CUA in pregnancy; therefore, outcomes were selected based on clinically recognized standards and prior epidemiological studies to ensure comparability and minimize reporting bias.

### 2.5. Covariates

Potential confounders were selected based on clinical relevance and the prior literature. Maternal age at delivery was included as a continuous variable, and parity was categorized as primiparous or multiparous. Pre-existing maternal comorbidities, including chronic hypertension and pregestational diabetes mellitus, were identified using ICD-10 codes prior to pregnancy.

To reduce residual confounding, the revised analysis additionally incorporated assisted reproductive technology (ART) usage. ART has been reimbursed nationally since October 2017 and is captured by procedure codes. To avoid over-adjustment, variables lying on the casual pathway between exposure and outcome, for example, PIH and GDM when modeling preterm birth, were treated as potential mediators and were not included as covariates in the corresponding models. The outcome-specific covariate sets are detailed in Supplementary Tables. Variables not available in the claims data, including body mass index and smoking status, could not be adjusted for.

### 2.6. Statistical Analysis

Baseline characteristics were summarized according to uterine anomaly status. Continuous variables were presented as means with standard deviations and compared using analysis of variance, whereas categorical variables were expressed as frequencies with percentages and compared using the chi-square test. Multivariable logistic regression models were used to estimate odds ratios (ORs) and 95% confidence intervals (CIs) for the association between uterine anomaly subtypes and each outcome, using women without uterine anomalies as the reference group. Adjusted models included maternal age, parity, chronic hypertension, and pregestational diabetes mellitus. To further explore differences across uterine anomaly subtypes, post hoc pairwise comparisons were performed with appropriate adjustment for multiple testing. All statistical analyses were conducted using a two-sided significance level of *p* < 0.05 and were performed using SAS version 9.4 (SAS Institute Inc., Cary, NC, USA).

To account for the multiple comparisons across outcomes and subtypes, post hoc comparisons between groups were performed with adjustment for multiple testing. To assess the robustness of the findings, we conducted three additional analyses: a sensitivity analysis restricted to women whose uterine anomaly was diagnosed before pregnancy (Table S1), a subgroup analysis restricted to nulliparous women (Table S2), and a sensitivity analysis restricted to pregnancies conceived without ART (Table S3). Because ART was substantially more frequent among women with CUA, its distribution across groups was additionally reported, and its potential confounding influence was examined through the ART excluded analysis. Post hoc pairwise comparisons between all group pairs were performed following the omnibus test, with adjustment for multiple comparisons; the adjusted pairwise *p*-values for all variables are provided in Table S4.

## 3. Results

A total of 878,000 women were included in the study. Among them, 871,962 had a normal uterus and 6038 had a CUA (double uterus, *n* = 3775; septate uterus, *n* = 657; bicornuate uterus, *n* = 1462; unicornuate uterus, *n* = 144). The incidence of CUA among pregnant women was 0.68% in this study. Other types of uterine anomaly (Q51.0, Q51.5, Q51.6, Q51.7, Q51.8, Q51.9) were excluded. Double uterus was the most common anomaly (62.5%), followed by bicornuate uterus (24.2%), septate uterus (10.9%), and unicornuate uterus (2.4%).

Significant differences in maternal and obstetric outcomes were observed across groups with and without CUA (Table 1). Maternal age at delivery was older in the septate, bicornuate, and unicornuate uterus groups. Women with CUA were more likely to be nulliparous than controls. Obstetric complications also notably increased, including placenta previa, placental abruption, and Cesarean delivery. In particular, the incidence of breech presentation was higher in the CUA groups (normal uterus 3.4%, double uterus 18.2%, septate uterus 16.0%, bicornuate uterus 14.2%, unicornuate uterus 29.2%).

Across the five groups, differences were statistically significant for most maternal and neonatal characteristics, as shown by the *p*-values reported in Table 1 and Table 2; PIH, GDM, PPH, neonatal male sex, NEC, and BPD did not differ significantly.

Table 2 shows the neonatal outcomes by CUA subtypes. The mean gestational age at birth was longer in the control group (normal uterus 38.9 weeks, double uterus 38.4 weeks, septate uterus 38.3 weeks, bicornuate uterus 38.6 weeks, unicornuate uterus 38.3 weeks). The preterm birth rate was significantly higher in the CUA groups (normal uterus 3.9%, double uterus 9.0%, septate uterus 9.1%, bicornuate uterus 7.5%, unicornuate uterus 6.2%). The incidence of SGA was higher in the CUA groups (normal uterus 10.3%, double uterus 15.6%, septate uterus 12.2%, bicornuate uterus 16.1%, unicornuate uterus 18.8%). Neonatal morbidity showed notable variation according to the presence and type of CUA.

Compared with women without uterine anomalies, the aOR for several obstetric and neonatal outcomes differed significantly according to the CUA subtype (Table 3). The Cesarean delivery rate was significantly higher across all CUA groups, with aORs ranging from 1.82 to 3.80 (all *p* < 0.001). The odds of breech presentation were markedly increased in all CUA subtypes (aOR 4.72–11.49, all *p* < 0.001). Placenta previa was also increased in double, septate, and bicornuate uterus (aOR 1.44–1.79, all *p* < 0.001). Placental abruption was elevated in double uterus (aOR 3.01, *p* < 0.001) and bicornuate uterus (aOR 2.03, *p* = 0.011), but not in other subtypes. No significant differences were observed in the rates of gestational hypertension or GDM across CUA subtypes. PPH showed a minimal increase limited to double uterus (aOR 1.10, *p* = 0.041).

**Figure 1:**
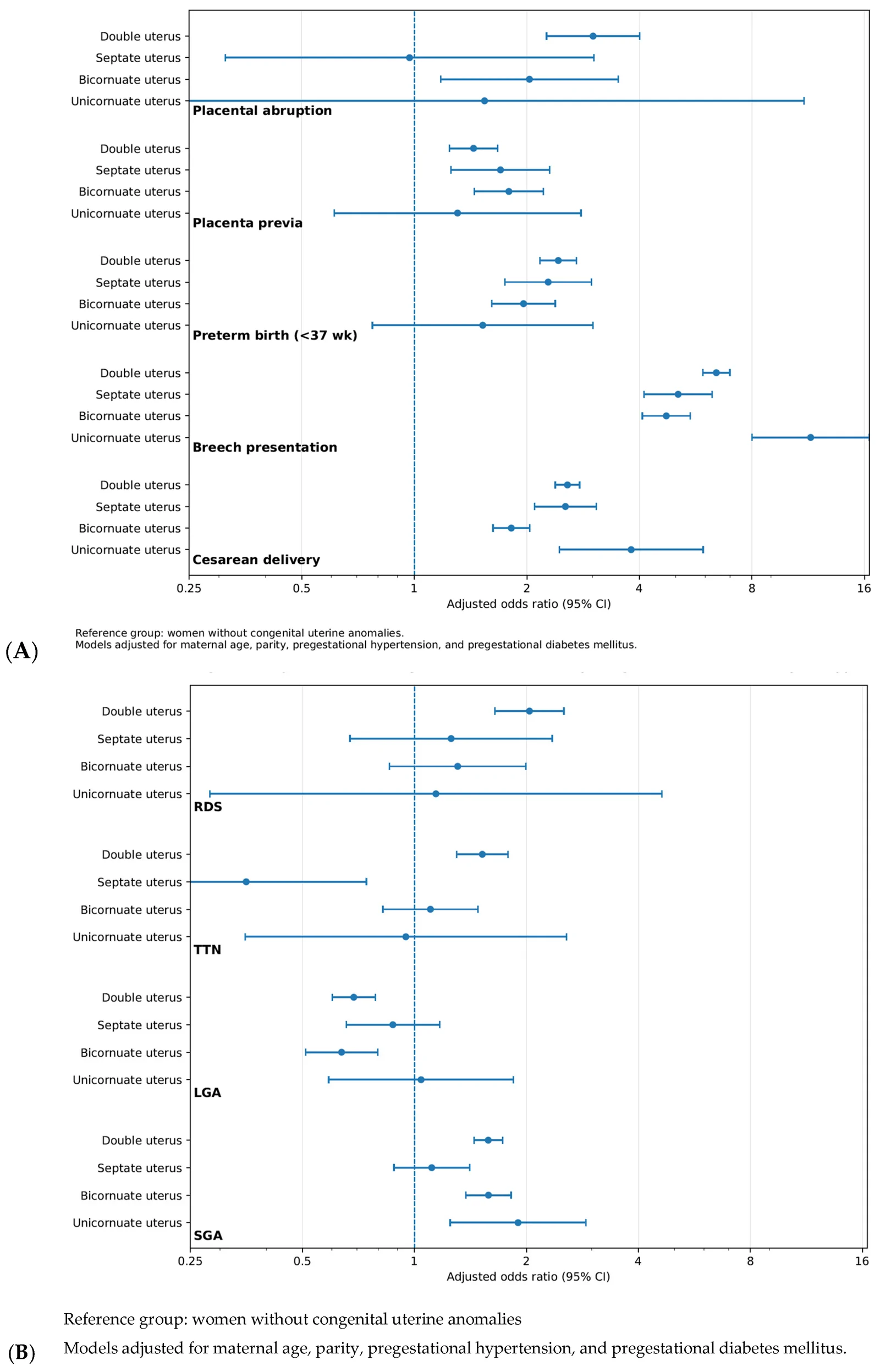
Adjusted odds ratios (95% confidence intervals) of (**A**) major obstetric outcomes and (**B**) neonatal outcomes by congenital uterine anomaly subtype, compared with women without congenital uterine anomalies. Models were adjusted for maternal age, parity, pre-pregnancy hypertension, and pre-pregnancy diabetes mellitus. In each panel, the dots represent the adjusted odds ratios (point estimates), the horizontal lines indicate the 95% confidence intervals, and the vertical dashed line denotes the null value (odds ratio = 1). RDS, respiratory distress syndrome; TTN, transient tachypnea of the newborn; LGA, large for gestational age; SGA, small for gestational age.

The odds of neonatal outcomes also differed among CUA types. Preterm birth (<37 weeks of gestation) was significantly more frequent in women with double, septate, and bicornuate uterus (aOR 1.96–2.43, all *p* < 0.001), whereas no significant association was observed in unicornuate uterus. The odds of SGA were increased in double, bicornuate, and unicornuate uterus (aOR 1.58–1.90). Neonatal respiratory morbidity was selectively increased: TTN was higher in double uterus (aOR 1.52; *p* < 0.001) but lower in septate uterus (aOR 0.35; *p* = 0.006), and RDS and IVH were increased only in double uterus (aOR 2.04 and 3.04, respectively; both *p* < 0.001). Other neonatal outcomes were not significantly associated. The key adjusted associations for obstetrics and neonatal outcomes are illustrated in Figure 1, whereas the complete numerical estimates are provided in Table 3. Other rare neonatal outcomes are reported in Supplementary Figure S2.

ART was markedly more common among women with CUA than controls, particularly in the septate (33.3%) and unicornuate (34.7%) groups compared with reference group (6.3%, Table 1). Nevertheless, the principal associations remained consistent across all additional analyses. When the analysis was restricted to women diagnosed before pregnancy (Table S1), to nulliparous women (Table S2), or to pregnancies conceived without ART (Table S3), the increased adjusted odds of Cesarean delivery (aOR 1.81–5.31, 2.23–3.10, and 1.81–4.50, respectively), breech presentation, preterm birth, and SGA persisted with similar magnitude and direction. These consistent results indicate that the observed associations were not explained by differences in the timing of diagnosis, parity imbalance, or the higher use of ART among women with CUA.

## 4. Discussion

This study provides data on the obstetric and neonatal outcomes of pregnant women with CUA. Considering the low incidence of CUA, this large-scale study shows that the risk of adverse obstetric outcomes in women with CUA is higher than in those with a normal uterus. Most women with CUA deliver at term, but they are at greater risk of preterm delivery, breech presentation, and Cesarean delivery than those with normal pregnancies. Especially, CUA was significantly associated with placental complications such as placenta previa and abruption, as well as neonatal morbidities including SGA, TTN, and RDS, particularly in cases of double uterus.

In this study, the most common type of CUA was double uterus, also described as didelphys in previous studies [5]. The incidence of uterine anomaly is unclear due to a lack of data and methodological bias. Previously, the literature reported that the prevalence of CUA varies from 0.06% to 38% [4,6]. Cunningham and colleagues noted a prevalence of CUA in the general population of 5%, and some researchers reported it to be as high as 38% [6,7,8,9,10,11,25,26]. The most common type of CUA seems to differ by race. In the United States, the most common type of CUA was bicornuate (73.5%), followed by arcuate (13.5%) and unicornuate (10.0%) [19]. In Korea, a previous study noted that CUA was found in one in 1326 patients (0.075%) [5]. The most common type was didelphys (30.6%), followed by bicornuate (19.4%), septate (9.1%), and unicornuate (8.6%). In this study, the prevalence of CUA was 0.68% in pregnant women, which is higher than in previous Korean studies but lower than global estimates.

Our subtype-specific estimates are broadly consistent with the direction of associations reported in previous smaller studies and meta-analyses, which have linked uterine anomalies with preterm birth, malpresentation, Cesarean delivery, and fetal growth restriction [13,19,20,27]. The main advance of the present study is the ability to quantify these risks separately for each anatomical subtype within a single ethnically homogeneous, nationwide cohort, which reduces the influence of between-study heterogeneity that has hampered comparison of earlier reports.

The risk of adverse pregnancy outcomes is higher in CUA pregnancies. The risks of obstetric outcomes such as placenta previa, placental abruption, fetal breech presentation, and Cesarean delivery were higher in CUA pregnancies. The pathophysiological mechanism for these adverse obstetric outcomes remains unclear. A decreased uterine volume may be a significant reason for adverse pregnancy outcomes. In a normal uterus, the average volume of the uterine cavity increases from 10 mL before pregnancy to 5000 mL at term [16]; in contrast, the uterine volume of an anomalous uterus is smaller than normal [17]. Decreased uterine cavity volume may increase the chance of abnormal placentation, cervical insufficiency, or placental insufficiency, leading to preterm labor and sometimes iatrogenic preterm delivery [14,15]. Neonatal outcomes were similar to those of other studies, in that preterm delivery and SGA were more common in CUA pregnancies [19,20,27]. The increased risk of SGA suggests that CUA may impose mechanical and vascular constraints on fetal growth caused by uteroplacental insufficiency. Neonatal morbidity showed a selective pattern, with the increased risk of TTN, RDS, and IVH observed primarily in double uterus. Although the precise mechanism remains unclear, the increased risk of neonatal respiratory complications in cases of double uterus may be attributable to restricted intrauterine space. Importantly, CUA was not associated with hypertensive disorders or GDM, suggesting that these conditions are more strongly connected to maternal systemic factors than to uterine structure.

These findings have several clinical implications. The elevated risks of breech presentation and Cesarean delivery support the need for enhanced antenatal surveillance and delivery planning including timely assessment of fetal presentation and counseling regarding mode of delivery. In addition, the increased risks of preterm birth and SGA highlight the importance of close monitoring of cervical length and fetal growth, and may inform decisions about the intensity of antenatal care in CUA pregnancies.

Moreover, our subtype-specific estimates can support individualized, evidence-based prenatal counseling. Women with double uterus may require more intensive monitoring for placental complications and neonatal respiratory morbidity, whereas for rare subtypes such as unicornuate uterus, the estimates are less reliable because of the small number of cases, and this should be made clear when counseling. These data may also help care team plan the timing and place of delivery.

The main strength of this study is its sample size, which is larger than those of previous studies. This study used a national big-data source, including more than 878,000 pregnant individuals and more than 6000 cases of CUA. This exceptionally large sample provided sufficient statistical power to examine not only overall associations but also rare maternal and neonatal outcomes and individual anatomical subtypes. Second, because the NHIS is a mandatory system covering approximately 97% of the Korean population, the cohort is nationally representative and largely free from the selection and loss of follow-up bias that can affect hospital-based samples. Third, previous studies included people of various races and could not account for racial differences in pregnancy outcomes; however, this study was conducted in a predominantly East Asian population, enabling effective control of potential confounders within a single ethnic group. Finally, the analyses were strengthened by adjustment for multiple potential confounders, correction for multiple comparisons, and a series of sensitivity and subgroup analyses, which together enhance the robustness and internal validity of the subtype-specific risk estimates.

This study also has several limitations. First, CUAs were identified solely from ICD-10 codes, which have not been formally validated for uterine anomalies within the NHIS, and the claims data do no record imaging or surgical confirmation. Some exposure misclassification is therefore inevitable, and women with suspected but unconfirmed anomalies may have been included. Second, the prevalence of this study was lower than other studies (4–7%) that reported systematic imaging of unselected populations, and women identified in our data who are likely to have undergone infertility evaluation of advanced imaging may represent a more symptomatic, severe end of the disease spectrum. This ascertainment and surveillance bias may have overestimated absolute risks. Third, although the models were adjusted for confounders, several clinically relevant variables such as body mass index, smoking history, and detailed obstetric history were unavailable and residual confounding cannot be excluded. Fourth, we lacked information on prior corrective surgeries and could not distinguish surgically treated from untreated septate uterus, which limits interpretation of the septate uterus findings. Fifth, spontaneous and medically indicated preterm births could not be reliably differentiated, so the excess of preterm birth may partly reflect indicated delivery. Sixth, the number of unicornuate uterus group was small, yielding side confidence intervals and low power. Finally, information on miscarriage and stillbirth was not available in this delivery-based cohort, and previous studies have shown associations between CUA and miscarriage and a higher prevalence of CUA among infertile women [14].

Future studies should seek to validate ICD-10 code-based case definitions of uterine anomalies against diagnostic modalities, and to assemble prospective cohorts with detailed information on anomaly subtype, prior corrective surgery, and confounders such as body mass index, smoking, and ART. Research linking anatomical severity to obstetric risk, evaluating the effect of interventions such as hysteroscopic septoplasty on metroplasty on subsequent pregnancy outcomes, and examining long-term neurodevelopmental and respiratory outcomes of infants born to mothers with CUA would be particularly valuable. Mechanism studies of placentation and uterine biomechanics in CUA as well as multicenter collaborations are also warranted to refine subtype-specific counseling.

## 5. Conclusions

Women with CUA are at increased risk of adverse pregnancy outcomes. Although the risk differs by CUA subtype, the risks of placenta previa, placental abruption, Cesarean delivery, malpresentation, and preterm birth are elevated, and neonates are at risk of low birth weight. Therefore, in prenatal counseling and tailored obstetric care, it is important for both clinicians and women with CUA to recognize that the likelihood of adverse outcomes is higher than in normal pregnancies.

## Figures and Tables

**Table 1:** Perinatal characteristics of women with a normal uterus versus congenital uterine anomalies.

Characteristic	Normal (*n* = 871,962)	Double Uterus (*n* = 3775)	Septate Uterus (*n* = 657)	Bicornuate Uterus (*n* = 1462)	Unicornuate Uterus (*n* = 144)	*p*-Value
Age (years)	33.2 ± 4.24	33.1 ± 4.1	35.4 ± 4.23	33.4 ± 4.1	34.1 ± 4.08	<0.0001
Nulliparity	483,067 (55.4%)	2272 (60.2%)	489 (74.4%)	983 (67.2%)	100 (69.4%)	<0.0001
DM	18,954 (2.2%)	83 (2.2%)	19 (2.9%)	36 (2.5%)	6 (4.2%)	0.0005
HTN	9433 (1.1%)	48 (1.3%)	15 (2.3%)	24 (1.6%)	3 (2.1%)	0.1101
PIH	48,473 (5.6%)	230 (6.1%)	40 (6.1%)	81 (5.5%)	13 (9.0%)	0.1742
GDM	98,706 (11.3%)	459 (12.2%)	97 (14.8%)	173 (11.8%)	25 (17.4%)	0.0043
Placenta previa	30,111 (3.5%)	185 (4.9%)	45 (6.9%)	91 (6.2%)	7 (4.9%)	<0.0001
PPH	109,776 (12.6%)	519 (13.8%)	88 (13.4%)	174 (11.9%)	20 (13.9%)	<0.0001
Placental abruption	3676 (0.4%)	48 (1.3%)	3 (0.5%)	13 (0.9%)	1 (0.7%)	0.5243
Cesarean delivery	469,631 (53.9%)	2829 (74.9%)	518 (78.8%)	1016 (69.5%)	120 (83.3%)	<0.0001
Breech presentation	29,268 (3.4%)	685 (18.2%)	105 (16.0%)	207 (14.2%)	42 (29.2%)	<0.0001
ART	54,555 (6.26%)	295 (7.81%)	219 (33.33%)	141 (9.64%)	50 (34.72%)	<0.0001

Values are presented as mean ± standard deviation or *n* (%). DM, diabetes mellitus; HTN, hypertension; PIH, pregnancy-induced hypertension; GDM, gestational diabetes mellitus; PPH, postpartum hemorrhage, ART; assisted reproductive technology. Overall *p*-values are shown. Adjusted pairwise comparisons between groups are provided in Table S4.

**Table 2:** Pregnancy outcomes and neonatal morbidities in the study population.

Outcome	Normal (*n* = 871,962)	Double Uterus (*n* = 3775)	Septate Uterus (*n* = 657)	Bicornuate Uterus (*n* = 1462)	Unicornuate Uterus (*n* = 144)	*p*-Value
Gestational age (weeks)	38.9 ± 1.43	38.4 ± 1.69	38.3 ± 1.71	38.6 ± 1.65	38.3 ± 1.47	<0.0001
Preterm birth (<37 weeks)	34,490 (4.0%)	341 (9.0%)	60 (9.1%)	110 (7.5%)	9 (6.3%)	<0.0001
Male sex	447,942 (51.4%)	1925 (51.0%)	363 (55.3%)	765 (52.3%)	63 (43.8%)	0.6402
Birth weight (kg)	3.20 ± 0.46	3.04 ± 0.51	3.06 ± 0.48	3.08 ± 0.54	3.01 ± 0.42	<0.0001
SGA	89,881 (10.3%)	590 (15.6%)	80 (12.2%)	235 (16.1%)	27 (18.8%)	<0.0001
LGA	76,158 (8.7%)	230 (6.1%)	50 (7.6%)	82 (5.6%)	13 (9.0%)	<0.0001
TTN	24,592 (2.82%)	160 (4.24%)	7 (1.07%)	46 (3.15%)	4 (2.78%)	0.0718
RDS	10,035 (1.15%)	87 (2.30%)	10 (1.52%)	22 (1.50%)	2 (1.39%)	<0.0001
NEC	496 (0.06%)	4 (0.11%)	0 (0%)	2 (0.14%)	0 (0%)	0.2276
IVH	1362 (0.16%)	18 (0.48%)	3 (0.46%)	3 (0.21%)	0 (0%)	0.0034
BPD	330 (0.04%)	1 (0.03%)	0 (0%)	2 (0.14%)	0 (0%)	0.2669

Values are presented as mean ± standard deviation or *n* (%). GA, gestational age; SGA, small for gestational age; LGA, large for gestational age; TTN, transient tachypnea of the newborn; RDS, respiratory distress syndrome; NEC, necrotizing enterocolitis; IVH, intraventricular hemorrhage; BPD, bronchopulmonary dysplasia. Overall *p*-values are shown. Adjusted pairwise comparisons between groups are provided in Table S4.

**Table 3:** Multivariable logistic regression analysis for pregnancy complications by congenital uterine anomaly subtype.

Outcome	Double Uterus aOR (95% CI)	*p*-Value	Septate Uterus aOR (95% CI)	*p*-Value	Bicornuate Uterus aOR (95% CI)	*p*-Value	Unicornuate Uterus aOR (95% CI)	*p*-Value
PIH	1.08 (0.94–1.24)	0.261	0.95 (0.69–1.31)	0.737	0.93 (0.74–1.17)	0.542	1.52 (0.86–2.70)	0.153
GDM	1.09 (0.99–1.20)	0.095	1.10 (0.88–1.37)	0.393	1.01 (0.86–1.19)	0.892	1.45 (0.93–2.25)	0.100
Placenta previa	1.44 (1.24–1.67)	<0.001	1.70 (1.25–2.30)	0.001	1.79 (1.45–2.22)	<0.001	1.31 (0.61–2.79)	0.493
Placental abruption	3.01 (2.26–4.01)	<0.001	0.97 (0.31–3.02)	0.959	2.03 (1.18–3.51)	0.011	1.54 (0.22–11.02)	0.667
PPH	1.10 (1.00–1.21)	0.041	1.06 (0.84–1.32)	0.637	0.93 (0.79–1.09)	0.353	1.11 (0.69–1.78)	0.674
Cesarean delivery	2.57 (2.38–2.77)	<0.001	2.54 (2.10–3.07)	<0.001	1.82 (1.62–2.03)	<0.001	3.80 (2.44–5.92)	<0.001
Breech presentation	6.42 (5.91–6.99)	<0.001	5.08 (4.12–6.26)	<0.001	4.72 (4.07–5.47)	<0.001	11.49 (8.01–16.47)	<0.001
Preterm birth (<37 weeks)	2.43 (2.17–2.72)	<0.001	2.28 (1.75–2.98)	<0.001	1.96 (1.61–2.38)	<0.001	1.52 (0.77–3.01)	0.224
SGA	1.58 (1.45–1.73)	<0.001	1.11 (0.88–1.41)	0.369	1.58 (1.38–1.82)	<0.001	1.90 (1.25–2.89)	0.003
LGA	0.69 (0.60–0.79)	<0.001	0.88 (0.66–1.17)	0.366	0.64 (0.51–0.80)	<0.001	1.04 (0.59–1.85)	0.889
TTN	1.52 (1.30–1.78)	<0.001	0.35 (0.17–0.74)	0.006	1.10 (0.82–1.48)	0.508	0.95 (0.35–2.56)	0.916
RDS	2.04 (1.65–2.52)	<0.001	1.26 (0.67–2.35)	0.477	1.31 (0.86–1.99)	0.214	1.14 (0.28–4.62)	0.852
NEC	1.87 (0.70–4.99)	0.215	—	—	2.40 (0.60–9.64)	0.217	—	—
IVH	3.04 (1.91–4.85)	<0.001	2.54 (0.82–7.92)	0.108	1.26 (0.41–3.92)	0.690	—	—
BPD	0.70 (0.10–5.00)	0.725	—	—	3.59 (0.89–14.45)	0.072	—	—

Reference group: women without congenital uterine anomalies. Models were adjusted for maternal age, parity, pre-pregnancy hypertension, and pre-pregnancy diabetes mellitus. aOR, adjusted odds ratio; CI, confidence interval; —, not estimable. Other abbreviations as in Table 1 and Table 2.

## Data Availability

The data presented in this study are available from the Korean National Health Insurance Service (NHIS) but restrictions apply to their availability; they were used under approval for the current study and are not publicly available. Data may be available from the authors upon reasonable request and with the permission of the NHIS.

## Supplementary Materials

The following supporting information can be downloaded at https://www.mdpi.com/article/10.3390/jcm15176788/s1, Figure S1. Flow diagram of study population selection (STROBE). Figure S2. Adjusted odds ratios of rare neonatal outcomes by congenital uterine anomaly subtypes. Table S1. Adjusted odds ratio restricted to women whose uterine anomaly was diagnosed before pregnancy. Table S2. Adjusted odds ratio among nulliparous women. Table S3. Adjusted odds ratio among pregnancies conceived without assisted reproductive technology. Table S4. Post hoc pairwise comparisons of baseline characteristics and outcomes among the groups.

## Abbreviations

The following abbreviations are used in this manuscript:

CUA	Congenital uterine anomaly
PIH	Pregnancy-induced hypertension
GDM	Gestational diabetes mellitus
PPH	Postpartum hemorrhage
ART	Assisted reproductive technology
SGA	Small for gestational age
LGA	Large for gestational age
TTN	Transient tachypnea of newborn
RDS	Respiratory distress syndrome
NEC	Necrotizing enterocolitis
IVH	Intraventricular hemorrhage
BPD	Bronchopulmonary dysplasia

## References

[1] Habiba M., Heyn R., Bianchi P., Brosens I., Benagiano G. (2021). The development of the human uterus: Morphogenesis to menarche. Hum. Reprod. Update.

[2] Pfeifer S.M., Attaran M., Goldstein J., Lindheim S.R., Petrozza J.C., Rackow B.W., Siegelman E., Troiano R., Winter T., Zuckerman A. (2021). ASRM müllerian anomalies classification 2021. Fertil. Steril..

[3] Dahiphale S.M., Potdar J., Acharya N., Jyotsna G., Desale R., Dahiphale S.M. (2024). Congenital anomalies of the female genital tract: A comprehensive review. Cureus.

[4] Chan Y.Y., Jayaprakasan K., Zamora J., Thornton J., Raine-Fenning N., Coomarasamy A. (2011). The prevalence of congenital uterine anomalies in unselected and high-risk populations: A systematic review. Hum. Reprod. Update.

[5] Jeon G.H., Park Y.R., Shin Y.J., Kim S.H., Chae H.D., Kim C.H., Kang B.M. (2010). Clinical characteristics of women with Müllerian anomaly: Twenty years of experience at Asan Medical Center. Korean J. Obstet. Gynecol..

[6] Simón C., Martinez L., Pardo F., Tortajada M., Pellicer A. (1991). Müllerian defects in women with normal reproductive outcome. Fertil. Steril..

[7] Makino T., Hara T., Oka C., Toyoshima K., Sugi T., Iwasaki K.-I., Umeuchi M., Iizuka R. (1992). Survey of 1120 Japanese women with a history of recurrent spontaneous abortions. Eur. J. Obstet. Gynecol. Reprod. Biol..

[8] Clifford K., Rai R., Watson H., Regan L. (1994). Pregnancy: An informative protocol for the investigation of recurrent miscarriage: Preliminary experience of 500 consecutive cases. Hum. Reprod..

[9] Acien P. (1996). Uterine anomalies and recurrent miscarriage. Infertil. Reprod. Med. Clin. N. Am..

[10] Homer H.A., Li T.-C., Cooke I.D. (2000). The septate uterus: A review of management and reproductive outcome. Fertil. Steril..

[11] Guimarães Filho H.A., Mattar R., Pires C.R., Araujo Júnior E., Moron A.F., Nardozza L.M. (2006). Prevalence of uterine defects in habitual abortion patients attended on at a university health service in Brazil. Arch. Gynecol. Obstet..

[12] Saravelos S.H., Cocksedge K.A., Li T.C. (2008). Prevalence and diagnosis of congenital uterine anomalies in women with reproductive failure: A critical appraisal. Hum. Reprod. Update.

[13] Venetis C.A., Papadopoulos S.P., Campo R., Gordts S., Tarlatzis B.C., Grimbizis G.F. (2014). Clinical implications of congenital uterine anomalies: A meta-analysis of comparative studies. Reprod. Biomed. Online.

[14] Carbonnel M., Pirtea P., de Ziegler D., Ayoubi J.M. (2021). Uterine factors in recurrent pregnancy losses. Fertil. Steril..

[15] Kim M.A., Kim H.S., Kim Y.H. (2021). Reproductive, obstetric and neonatal outcomes in women with congenital uterine anomalies: A systematic review and meta-analysis. J. Clin. Med..

[16] Myers K.M., Elad D. (2017). Biomechanics of the human uterus. Wiley Interdiscip. Rev. Syst. Biol. Med..

[17] Negm S.M., Kamel R.A., El-Zayat H.A., Elbigawy A.F., El-Toukhy M.M., Amin A.H., Nicolaides K.H. (2022). The value of three-dimensional ultrasound in identifying Mullerian anomalies at risk of adverse pregnancy outcomes. J. Matern.-Fetal Neonatal Med..

[18] Fedele F., Bulfoni A., Parazzini F., Levi-Setti P.E., Busnelli A. (2024). Assisted reproductive technology outcomes in women with congenital uterine anomalies: A systematic review. Arch. Gynecol. Obstet..

[19] Mandelbaum R.S., Anderson Z.S., Masjedi A.D., Violette C.J., McGough A.M., Doody K.A., Guner J.Z., Quinn M.M., Paulson R.J., Ouzounian J.G. (2024). Obstetric outcomes of women with congenital uterine anomalies in the United States. Am. J. Obstet. Gynecol. MFM.

[20] Zhang Y., Jie Q. (2010). Obstetric outcome of women with uterine anomalies in China. Chin. Med. J..

[21] Kim J.A., Kim J., Roh E., Hong S.-H., Lee Y.-B., Baik S.H., Choi K.M., Noh E., Hwang S.Y., Cho G.J. (2020). Association of fasting plasma glucose variability with gestational diabetes mellitus: A nationwide population-based cohort study. BMJ Open Diabetes Res. Care.

[22] Jung Y.M., Oh G.C., Noh E., Lee H.-Y., Oh M.-J., Park J.S., Jun J.K., Lee S.M., Cho G.J. (2022). Pre-pregnancy blood pressure and pregnancy outcomes: A nationwide population-based study. BMC Pregnancy Childbirth.

[23] Koo B.K., Lee J.H., Kim J., Jang E.J., Lee C.-H. (2016). Prevalence of Gestational Diabetes Mellitus in Korea: A National Health Insurance Database Study. PLoS ONE.

[24] Lee J.K., Jang H.L., Kang B.H., Lee K.-S., Choi Y.-S., Shim K.S., Lim J.W., Bae C.-W., Chung S.-H. (2016). Percentile Distributions of Birth Weight according to Gestational Ages in Korea (2010–2012). J. Korean Med. Sci..

[25] Cunningham F., Leveno K.J., Bloom S.L., Spong C.Y., Dashe J.S., Hoffman B.L., Casey B.M., Sheffield J.S. (2018). Physiology of labor. Williams Obstetrics.

[26] Guimarães Filho H.A., Mattar R., Pires C.R., Júnior E.A., Moron A.F., Nardozza L.M.M. (2006). Comparison of hysterosalpingography, hysterosonography and hysteroscopy in evaluation of the uterine cavity in patients with recurrent pregnancy losses. Arch. Gynecol. Obstet..

[27] Hua M., Odibo A.O., Longman R.E., Macones G.A., Roehl K.A., Cahill A.G. (2011). Congenital uterine anomalies and adverse pregnancy outcomes. Am. J. Obstet. Gynecol..

