# Supplementary material for: Congenital Uterine Anomalies and Pregnancy Outcomes: A Population-Based Retrospective Cohort Study
Source: J Clin Med. 2026 Sep 1;15(17):6788. doi: 10.3390/jcm15176788 (PMC13565960; doi:10.3390/jcm15176788)
Supplement: Supplementary file 1 [file jcm-15-06788-s001.zip › jcm-4423616-supplementary.pdf]

Journal of Clinical Medicine

Supplementary

Figure S1. Flow diagram of study population selection (STROBE).

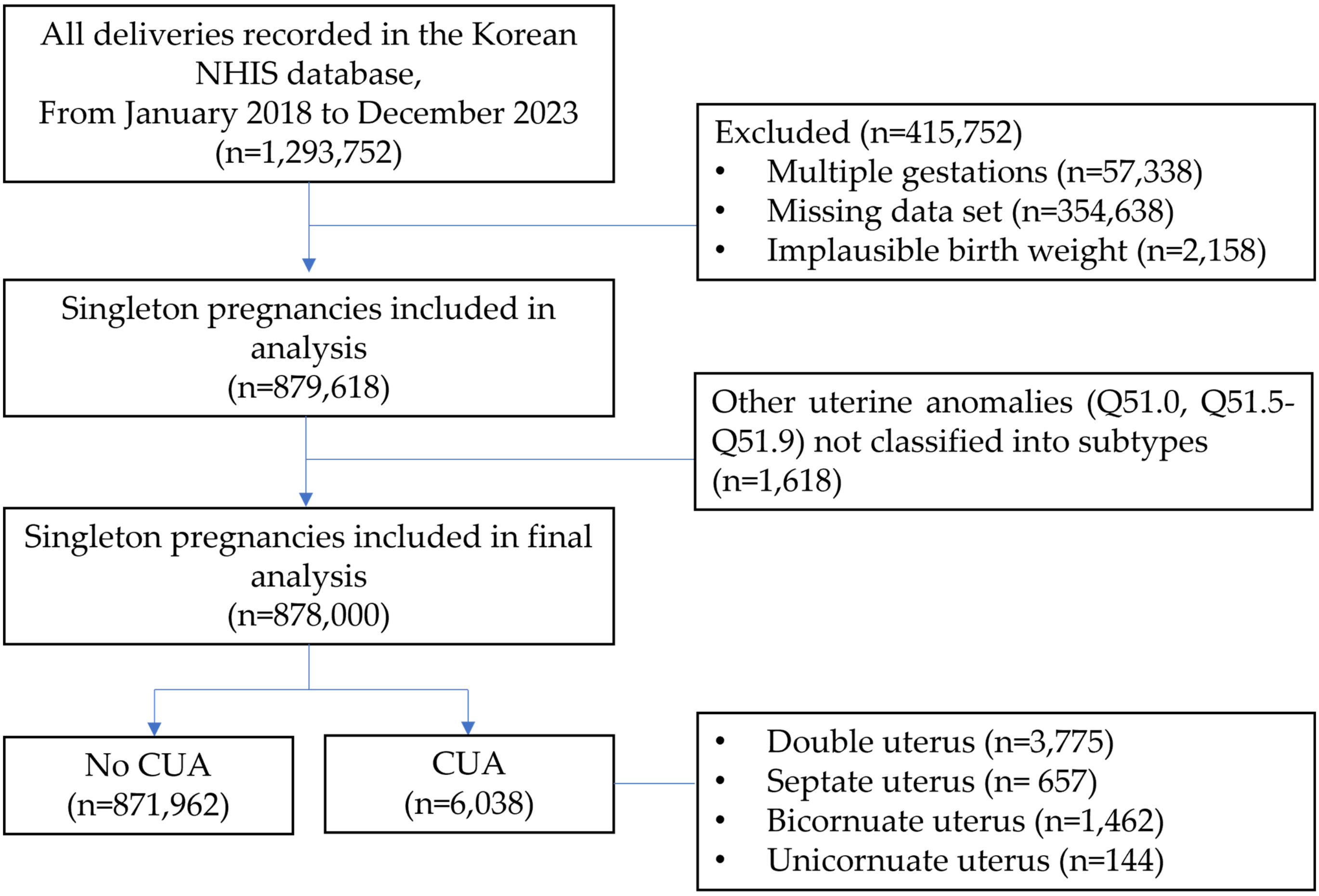

Figure S2. Adjusted odds ratios of rare neonatal outcomes by congenital uterine anomaly subtypes.

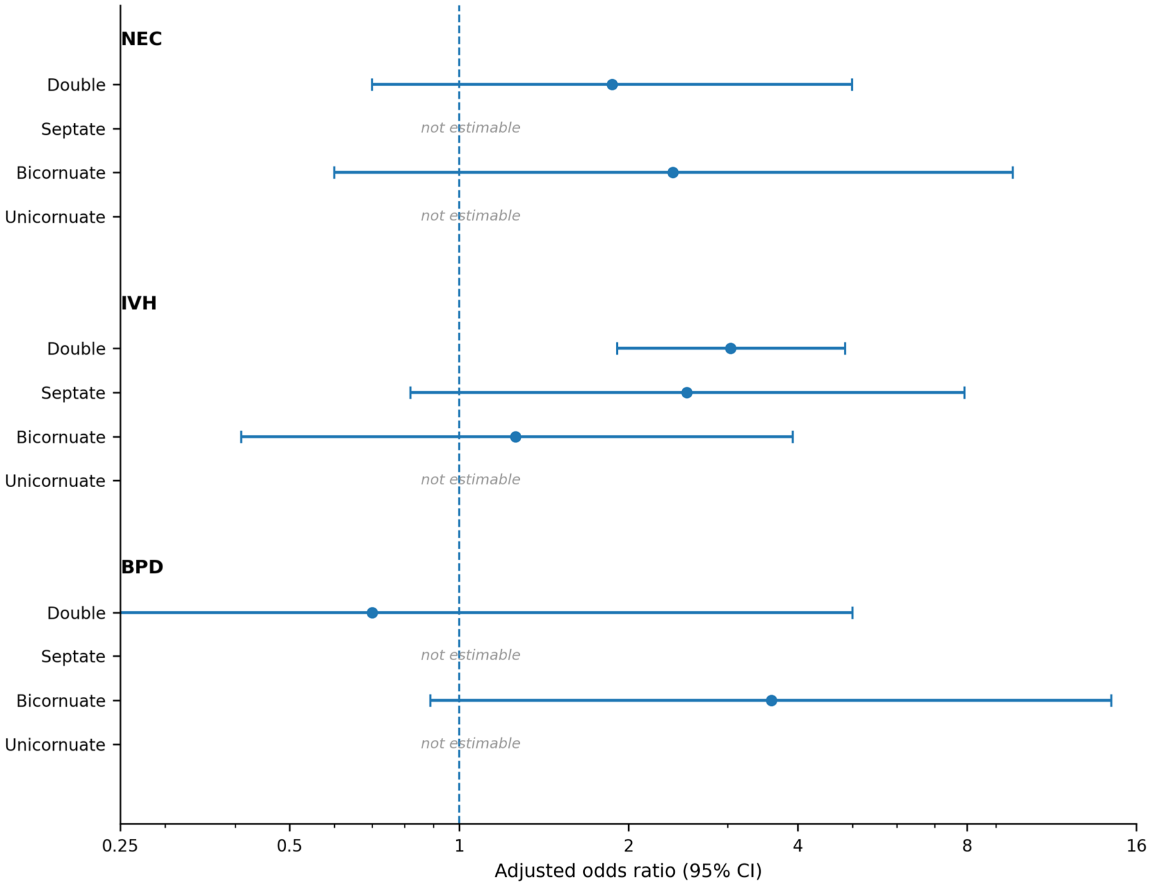

Reference group: women without congenital uterine anomalies. Models adjusted for maternal age, parity, pre-pregnancy hypertension, and pre-pregnancy diabetes mellitus.

Table S1. Adjusted odds ratio restricted to women whose uterine anomaly was diagnosed before pregnancy

Outcome	Double aOR (95% CI)	p	Septate aOR (95% CI)	p	Bicornuate aOR (95% CI)	p	Unicornuate aOR (95% CI)	p
IUI	1.04 (0.73–1.49)	0.820	0.60 (0.08–4.33)	0.615	0.84 (0.44–1.63)	0.612	NE	0.885
ART	1.12 (0.94–1.33)	0.222	1.29 (0.64–2.58)	0.473	0.84 (0.61–1.17)	0.312	7.83 (4.49–13.65)	<0.001
PIH	1.23 (1.04–1.45)	0.017	1.06 (0.49–2.28)	0.879	1.02 (0.75–1.38)	0.914	2.46 (1.18–5.15)	0.017
GDM	1.03 (0.90–1.18)	0.647	1.41 (0.85–2.35)	0.182	0.99 (0.79–1.24)	0.940	1.21 (0.59–2.47)	0.597
Placenta previa	1.46 (1.20–1.77)	<0.001	0.98 (0.36–2.65)	0.967	1.62 (1.19–2.22)	0.002	0.86 (0.21–3.53)	0.836
Placental abruption	3.46 (2.44–4.92)	<0.001	NE	0.925	2.46 (1.22–4.94)	0.011	NE	0.939
PPH	1.12 (0.99–1.26)	0.061	0.99 (0.58–1.70)	0.975	0.95 (0.77–1.18)	0.656	0.34 (0.11–1.07)	0.066
Cesarean delivery	2.35 (2.14–2.58)	<0.001	2.88 (1.89–4.41)	<0.001	1.81 (1.55–2.10)	<0.001	5.31 (2.62–10.75)	<0.001
Breech presentation	6.73 (6.04–7.49)	<0.001	8.06 (5.22–12.46)	<0.001	4.94 (4.05–6.04)	<0.001	13.61 (8.08–22.92)	<0.001
Preterm birth (<37 weeks)	2.22 (1.91–2.58)	<0.001	2.99 (1.68–5.33)	<0.001	1.86 (1.41–2.45)	<0.001	0.38 (0.05–2.74)	0.337
SGA	1.76 (1.57–1.97)	<0.001	1.74 (1.07–2.81)	0.024	1.95 (1.63–2.34)	<0.001	2.88 (1.64–5.06)	<0.001
LGA	0.63 (0.53–0.76)	<0.001	0.36 (0.13–0.98)	0.045	0.64 (0.48–0.87)	0.004	0.85 (0.34–2.13)	0.737
TTN	1.53 (1.24–1.88)	<0.001	0.89 (0.28–2.79)	0.837	1.15 (0.77–1.71)	0.502	1.66 (0.52–5.29)	0.392
RDS	1.93 (1.45–2.57)	<0.001	1.47 (0.36–5.95)	0.589	0.89 (0.45–1.80)	0.754	1.34 (0.18–9.65)	0.773
NEC	1.58 (0.40–6.36)	0.516	NE	0.986	NE	0.964	NE	0.990
IVH	3.83 (2.21–6.62)	<0.001	5.42 (0.76–38.83)	0.093	0.83 (0.12–5.88)	0.849	NE	0.958

BPD	1.21 (0.17–8.61)	0.850	NE	0.987	NE	0.966	NE	0.991

Odds ratios were adjusted for maternal age, parity, pre-pregnancy hypertension, and pre-pregnancy diabetes mellitus. The reference group was women without a congenital uterine anomaly. aOR, adjusted odds ratio; CI, confidence interval; IUI, intrauterine insemination; ART, assisted reproductive technology; NE, not estimable owing to small cell counts (data separation). Other abbreviations as in Tables 1 and 2.

Table S2. Adjusted odds ratio among nulliparous women.

Subgroup analysis restricted to nulliparous women.

Outcome	Double aOR (95% CI)	p	Septate aOR (95% CI)	p	Bicornuate aOR (95% CI)	p	Unicornuate aOR (95% CI)	p
IUI	0.95 (0.70–1.29)	0.750	1.17 (0.71–1.93)	0.537	1.01 (0.65–1.55)	0.977	1.31 (0.41–4.15)	0.647
ART	1.27 (1.10–1.46)	0.001	4.51 (3.69–5.50)	<0.001	1.43 (1.17–1.75)	<0.001	7.64 (4.93–11.84)	<0.001
PIH	1.10 (0.94–1.30)	0.233	1.00 (0.71–1.43)	0.978	1.00 (0.77–1.29)	0.991	1.73 (0.92–3.24)	0.089
GDM	1.12 (0.98–1.27)	0.084	1.15 (0.90–1.47)	0.271	0.97 (0.80–1.19)	0.776	1.23 (0.70–2.14)	0.476
Placenta previa	1.32 (1.09–1.61)	0.005	1.80 (1.29–2.51)	<0.001	1.83 (1.43–2.36)	<0.001	1.02 (0.38–2.79)	0.963
Placental abruption	2.36 (1.59–3.51)	<0.001	0.80 (0.20–3.21)	0.753	1.93 (1.00–3.72)	0.051	2.06 (0.29–14.78)	0.472
PPH	1.13 (1.00–1.27)	0.041	1.04 (0.81–1.35)	0.740	0.96 (0.80–1.16)	0.681	1.18 (0.68–2.04)	0.558
Cesarean delivery	2.38 (2.16–2.63)	<0.001	2.23 (1.78–2.79)	<0.001	1.59 (1.38–1.82)	<0.001	3.10 (1.83–5.25)	<0.001
Breech presentation	6.08 (5.50–6.71)	<0.001	3.74 (2.97–4.72)	<0.001	3.81 (3.21–4.52)	<0.001	9.85 (6.50–14.95)	<0.001
Preterm birth (<37 weeks)	2.68 (2.32–3.09)	<0.001	2.12 (1.54–2.92)	<0.001	1.92 (1.50–2.45)	<0.001	1.79 (0.83–3.88)	0.140
SGA	1.64 (1.48–1.82)	<0.001	1.16 (0.90–1.49)	0.268	1.60 (1.36–1.88)	<0.001	2.02 (1.26–3.24)	0.004
LGA	0.64 (0.53–0.77)	<0.001	1.05 (0.76–1.45)	0.768	0.53 (0.39–0.72)	<0.001	0.76 (0.33–1.72)	0.505
TTN	1.49 (1.21–1.83)	<0.001	0.47 (0.22–0.99)	0.047	0.92 (0.62–1.35)	0.664	1.02 (0.32–3.23)	0.969
RDS	2.41 (1.85–3.15)	<0.001	1.36 (0.68–2.74)	0.391	1.40 (0.84–2.34)	0.194	1.75 (0.43–7.13)	0.433
NEC	3.23 (1.20–8.69)	0.020	NE	0.972	1.84 (0.26–13.12)	0.543	NE	0.987
IVH	2.67 (1.43–4.98)	0.002	2.15 (0.54–8.66)	0.280	0.59 (0.08–4.23)	0.604	NE	0.966
BPD	1.31 (0.18–9.34)	0.789	NE	0.981	NE	0.974	NE	0.991

Odds ratios were adjusted for maternal age, pre-pregnancy hypertension, and pre-pregnancy diabetes mellitus (parity was not included because the analysis was restricted to nulliparous women). Abbreviations as in Table S1.

Table S3. Adjusted odds ratio among pregnancies conceived without assisted reproductive technology.

Sensitivity analysis restricted to non-ART conceptions

Outcome	Double aOR (95% CI)	p	Septate aOR (95% CI)	p	Bicornuate aOR (95% CI)	p	Unicornuate aOR (95% CI)	p
IUI	1.06 (0.80–1.40)	0.684	1.90 (1.15–3.15)	0.013	1.32 (0.90–1.95)	0.151	2.78 (1.00–7.68)	0.049
PIH	1.09 (0.95–1.25)	0.232	0.86 (0.57–1.32)	0.499	0.94 (0.74–1.19)	0.586	1.71 (0.85–3.42)	0.131
GDM	1.06 (0.95–1.17)	0.314	0.97 (0.73–1.30)	0.856	1.01 (0.85–1.20)	0.907	1.53 (0.88–2.65)	0.132
Placenta previa	1.50 (1.28–1.76)	<0.001	1.10 (0.68–1.79)	0.692	1.86 (1.48–2.35)	<0.001	1.30 (0.48–3.54)	0.609
Placental abruption	3.08 (2.28–4.16)	<0.001	NE	0.927	2.33 (1.35–4.04)	0.002	NE	0.966
PPH	1.10 (1.00–1.22)	0.044	0.93 (0.70–1.24)	0.625	0.93 (0.79–1.10)	0.410	0.92 (0.49–1.73)	0.795
Cesarean delivery	2.56 (2.37–2.76)	<0.001	2.80 (2.23–3.52)	<0.001	1.81 (1.61–2.04)	<0.001	4.50 (2.58–7.85)	<0.001
Breech presentation	NE	<0.001	NE	<0.001	NE	<0.001	1.06 (1.05–1.06)	<0.001

Preterm birth (<37 weeks)	2.37 (2.10–2.67)	<0.001	2.33 (1.67–3.26)	<0.001	1.98 (1.60–2.44)	<0.001	1.66 (0.72–3.81)	0.234
SGA	1.60 (1.46–1.76)	<0.001	1.10 (0.82–1.47)	0.516	1.61 (1.39–1.86)	<0.001	2.28 (1.39–3.74)	0.001
LGA	0.69 (0.59–0.79)	<0.001	0.92 (0.65–1.30)	0.626	0.63 (0.50–0.80)	<0.001	0.96 (0.47–2.00)	0.924
TTN	1.41 (1.19–1.68)	<0.001	0.47 (0.21–1.05)	0.066	1.05 (0.77–1.45)	0.743	1.12 (0.35–3.54)	0.846
RDS	1.97 (1.56–2.48)	<0.001	1.20 (0.54–2.68)	0.660	1.23 (0.77–1.97)	0.378	1.85 (0.45–7.53)	0.391
NEC	1.58 (0.51–4.91)	0.432	NE	0.961	2.78 (0.69–11.17)	0.149	NE	0.982
IVH	2.92 (1.75–4.86)	<0.001	2.86 (0.71–11.50)	0.138	1.50 (0.48–4.66)	0.485	NE	0.952
BPD	0.82 (0.12–5.86)	0.845	NE	0.968	4.34 (1.08–17.45)	0.039	NE	0.984

Odds ratios were adjusted for maternal age, parity, pre-pregnancy hypertension, and pre-pregnancy diabetes mellitus. ART is not applicable because these pregnancies were conceived without ART. Breech presentation was not reliably estimable owing to data separation; the unadjusted odds remained markedly elevated across subtypes. Abbreviations as in Table S1.

Table S4. Post hoc pairwise comparisons of baseline characteristics and outcomes among the groups.

Variable	Overall p	N vs D	N vs S	N vs B	N vs U	D vs S	D vs B	D vs U	S vs B	S vs U	B vs U
Maternal age	<0.001	0.842	<0.001	0.421	0.130	<0.001	0.256	0.088	<0.001	0.036	0.431
Nulliparity	<0.001	<0.001	<0.001	0.307	<0.001	0.050	0.880	<0.001	0.992	<0.001	0.021
PIH	0.174	1.000	0.962	0.685	0.729	0.992	0.746	0.986	0.552	1.000	0.509
GDM	0.004	0.436	0.999	0.443	0.621	0.423	0.939	0.101	0.408	0.984	0.264
HTN	<0.001	0.254	0.854	0.931	0.868	0.784	1.000	0.066	0.993	0.372	0.854
DM	0.110	0.868	0.987	0.640	1.000	0.983	0.924	0.810	0.774	0.966	0.611
Placenta previa	<0.001	0.174	0.238	1.000	<0.001	0.971	0.845	<0.001	0.948	<0.001	0.931
Placental abruption	<0.001	0.067	0.458	0.896	<0.001	0.736	0.997	1.000	0.998	0.111	0.993
PPH	0.524	1.000	0.514	1.000	0.333	0.922	1.000	0.984	0.976	0.960	0.994
Cesarean delivery	<0.001	0.488	0.013	0.414	<0.001	0.003	0.916	<0.001	0.038	<0.001	<0.001
Breech presentation	<0.001	0.096	<0.001	<0.001	<0.001	0.338	<0.001	<0.001	<0.001	<0.001	<0.001
IUI	0.003	0.294	0.753	0.794	0.996	0.879	1.000	0.153	0.963	0.464	0.740
ART	<0.001	<0.001	0.200	<0.001	0.004	<0.001	0.984	<0.001	<0.001	<0.001	<0.001
Gestational age	<0.001	<0.001	<0.001	<0.001	<0.001	0.838	<0.001	0.989	0.003	1.000	0.331
Male sex	0.640	0.398	0.945	0.572	0.995	0.817	0.181	0.412	0.425	0.970	0.501
Birth weight	<0.001	<0.001	<0.001	<0.001	<0.001	0.925	0.097	0.961	0.913	0.855	0.547
SGA	<0.001	0.126	0.994	0.834	<0.001	0.115	0.239	0.650	0.908	<0.001	0.026
LGA	<0.001	0.805	0.989	0.826	<0.001	0.684	0.990	0.903	0.749	0.001	1.000
TTN	0.072	<0.001	0.334	0.898	<0.001	0.128	0.868	0.118	1.000	0.967	1.000
RDS	<0.001	0.558	0.207	0.907	<0.001	1.000	1.000	0.940	1.000	0.809	0.999
NEC	0.228	0.894	0.996	0.992	0.812	0.829	1.000	0.985	0.980	0.803	0.999
IVH	0.003	1.000	0.294	0.736	<0.001	0.769	0.816	0.440	0.986	0.994	0.994
BPD	0.267	0.999	0.496	1.000	0.998	0.692	1.000	0.993	0.958	0.438	1.000

Adjusted pairwise p-values following the omnibus test. N, normal uterus; D, double uterus; S, septate uterus; B, bicornuate uterus; U, unicornuate uterus.Overall p-values were derived from one-way analysis of variance (continuous variables) or the chi-square test (categorical variables). Pairwise p-values were adjusted for multiple comparisons. Abbreviations as in Table S1.
